# When COVID-19 sits on people's laps: A systematic review of SARS-CoV-2 infection prevalence in household dogs and cats

**DOI:** 10.1016/j.onehlt.2023.100497

**Published:** 2023-02-03

**Authors:** Ruoshui Guo, Cecilia Wolff, Joaquin M. Prada, Lapo Mughini-Gras

**Affiliations:** a Utrecht University, Utrecht, the Netherlands; b Norwegian Veterinary Institute (NVI), Ås, Norway; c University of Surrey, Guildford, United Kingdom; d National Institute for Public Health and the Environment (RIVM), Bilthoven, the Netherlands

**Keywords:** COVID-19, Pets, Prevalence, SARS-CoV-2, Transmission

## Abstract

During the COVID-19 pandemic, questions were raised about whether SARS-CoV-2 can infect pets and the potential risks posed to and by their human owners. We performed a systematic review of studies on SARS-CoV-2 infection prevalence in naturally infected household dogs and cats conducted worldwide and published before January 2022. Data on SARS-CoV-2 infection prevalence, as determined by either molecular or serological methods, and accompanying information, were summarized. Screening studies targeting the general dog or cat populations were differentiated from those targeting households with known COVID-19-positive people. Studies focusing on stray, sheltered or working animals were excluded. In total, 17 studies were included in this review. Fourteen studies investigated cats, 13 investigated dogs, and 10 investigated both. Five studies reported molecular prevalence, 16 reported seroprevalence, and four reported both. All but two studies started and ended in 2020. Studies were conducted in eight European countries (Italy, France, Spain, Croatia, Germany, the Netherlands, UK, Poland), three Asian countries (Iran, Japan, China) and the USA. Both molecular and serological prevalence in the general pet population were usually below 5%, but exceeded 10% when COVID-19 positive people were known to be present in the household. A meta-analysis provided pooled seroprevalence estimates in the general pet population: 2.75% (95% Confidence Interval [CI]: 1.56-4.79%) and 0.82% (95% CI: 0.26-2.54%) for cats and dogs, respectively. This review highlighted the need for a better understanding of the possible epizootic implications of the COVID-19 pandemic, as well as the need for global standards for SARS-CoV-2 detection in pets.

## Highlights

• We summarized SARS-CoV-2 prevalence data in naturally infected pet dogs and cats.
• Most studies reported seroprevalence and were conducted in Europe in 2020.
• Molecular and serological prevalence in screening studies was generally below 5%.
• Prevalence exceeded 10% when pets were exposed to known COVID-19 positive owners.
• Epizootic implications of SARS-CoV-2 and testing standards for pets need more attention.

## 1 Introduction

The Coronavirus Disease 2019 (COVID-19) is a respiratory disease caused by the Severe Acute Respiratory Syndrome Coronavirus 2 (SARS-CoV-2), a positive-sense single-stranded RNA virus that appeared among humans in 2019 and has had an enormous health, social and economic impact globally ever since [1]. Four structural proteins of SARS-CoV-2 are the spike (S), envelope (E), membrane (M), and nucleocapsid (N) proteins [2]. The S protein is the ‘key’ to entering host cells and has two subunits, S1 and S2, with S1 being the receptor-binding domain (RBD) and S2 being involved membrane fusion. Detection methods used in several SARS-CoV-2 prevalence studies are based on the presence of two subunits [[3], [4], [5]].

It is thought that the first spillover of SARS-CoV-2 occurred from wild animals to humans in Wuhan, China, although the true origin of the virus is still under debate [6]. Yet, a growing body of evidence has shown that several animal species can be naturally infected with SARS-CoV-2, including but not limited to dogs, cats, tigers, lions and minks, whereas farm animals like pigs, chickens and ducks are far less susceptible to SARS-CoV-2 infection [7]. Among companion animals, cats seem to be more susceptible to SARS-CoV-2 infection than dogs, with the virus being able to replicate more efficiently and be transmitted through aerosols in cats [8]. Therefore, questions have been raised about whether dogs and cats represent a source of SARS-CoV-2 infection for humans, although several studies have emphasized that the direction of transmission could also be from humans to their pets [9]. While the exact transmission dynamics remain unclear, SARS-CoV-2 transmission among pets, and between pets and humans, has been observed [[10], [11], [12]].

SARS-CoV-2 infected cats and dogs are mostly asymptomatic, contrary to humans that often develop respiratory symptoms [[13], [14], [15]]. Yet, asymptomatic dogs and cats are still able to shed the virus and therefore contribute to its spread [16]. Occasionally, dogs and cats might show symptoms upon SARS-CoV-2 infection, particularly cats have been reported to develop both respiratory and digestive symptoms [17].

With the main transmission route being the exposure to respiratory droplets and with an incubation period of up to 14 days [18], SARS-CoV-2 can spread rapidly in the human population. Indeed, after only three months from the first reports of human cases in December 2019, COVID-19 was declared a global pandemic by the World Health Organization (WHO). Consequently, household dogs and cats have also been increasingly exposed to SARS-CoV-2, as often they cannot avoid viral exposure, similar to any other household member. Some studies have assessed the association between COVID-19 occurrence among people and positivity for SARS-CoV-2 infection among their household pets, showing that pet owners with COVID-19 represent a significant risk factor for SARS-CoV-2 positivity among their dogs and cats [4,[19], [20], [21]].

Keeping dogs or cats as pets in a household is very common in several countries. For instance, it has been reported that 56% of Canadian households had at least one dog or cat in 2009 [22]. Similarly, 47% of Italian households owned at least one dog in 2020 [23], 62% of British households owned at least one cat in 2015 [24], and pet ownership in the 25 metropolitan areas of the USA ranged from 26% to 60% in 2018 [25]. These numbers entail considerable opportunities for close human-pet interaction. Given the high number of cats and dogs cohabiting with their human owners worldwide and the possibility for SARS-CoV-2 to infect both pets and humans, it seems relevant to monitor SARS-CoV-2 prevalence among pets also for public health purposes. Moreover, natural SARS-CoV-2 infection in household cats and dogs has implications for potential zoonotic transmission also for other SARS-related outbreaks in the future.

Next to One Health surveillance systems, literature reviews can provide valuable information about the prevalence of SARS-CoV-2 infection in different populations. Two recent reviews focused on SARS-CoV-2 infection prevalence in animals [26,27]. One of them presented descriptively SARS-CoV-2 infection prevalence estimates in selected companion animal populations [26], while the other one used a systematic review and meta-analysis approach to summarize SARS-CoV-2 infection prevalence in animals based on molecular detection and serology among 32 published studies, with the main limitation being the lack of differentiation between studies on SARS-CoV-2 infection prevalence in screenings and in outbreaks in closed groups of animals [27]. Other reviews reporting SARS-CoV-2 infection prevalence estimates included humans, animals and the environment in specific countries [28] or focused on SARS-CoV-2 transmission between humans and other animals [29,30]. Yet, household dogs or cats were hardly the specific focus of this type of reviews. Moreover, the COVID-19 pandemic has evolved through different phases, which can be expected to have had an effect on SARS-CoV-2 circulation among pets as well. Therefore, the aim of this systematic review was to summarize the available SARS-CoV-2 infection prevalence data in naturally infected dogs and cats kept as pets, taking into account factors such as the study period and country, SARS-CoV-2 detection method and sample type.

## 2 Methods

### 2.1 Literature search and selection

A two-step literature search and selection procedure was performed consisting of a primary search and selection and a re-search and re-selection. Firstly, we searched relevant literature in Pubmed, EMBASE, PsycINFO, medRxiv and bioRxiv using the COAP database (https://ispmbern.github.io/covid-19/living-review/) to identify both published and preprinted COVID-19 related studies. The search was conducted in January 2022 using the search string ‘(prevalence) and ((COVID) or (corona)) and ((pets) or (cats) or (felines) or (dogs) or (canines))’ in titles and abstracts from the COAP database. These studies were then manually screened to filter out any repeated or COVID-19 unrelated publication, after which a shortlist was obtained. In the re-search and re-selection step, we evaluated the shortlisted publications and manually retrieved their cited references to reach concept saturation. Both selection and re-selection of publications were based on the following two criteria: 1) the study population included domestic (household) cats and/or dogs kept as pets for companionship purposes (i.e., studies focusing exclusively on stray, sheltered or working animals were excluded); the animals recruited at veterinary clinics, laboratories or other veterinary facilities were considered as pets if not specified otherwise; (2) the study differentiated between prevalence derived from general screenings and from known COVID-19 positive households (but excluding those with only a suspicion). Two authors independently screened the studies and any discrepancy was resolved through a consensus discussion.

**Fig. 1:**
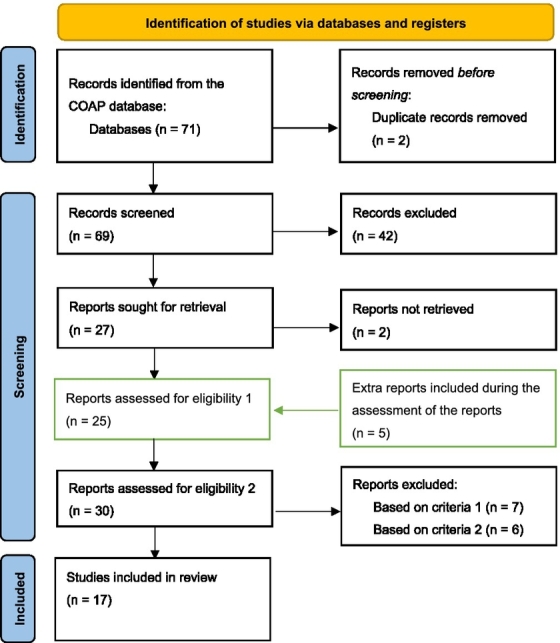
Flow chart of the literature search and study selection process based on the PRISMA 2020 Standard for Systematic Reviews.

The methods used in this review followed the Preferred Reporting Items for Systematic reviews and Meta-Analyses, extension for Scoping Reviews (PRISMA-ScR) guidelines [31]. A flowchart summarizing the literature search and selection is reported in Fig. 1.

### 2.2 Data extraction

Data on prevalence, as determined using either molecular or serological methods, and accompanying information, were extracted from each selected study. Data were manually extracted from text, tables, figures or supplementary files. The accompanying information included the animal species, study period and country, SARS-CoV-2 detection method, sample type, the total number of tested animals and the corresponding number of positive animals, as well as the study-provided percent prevalence and respective 95% confidence intervals (95% CI). To compare prevalence estimates of dogs and cats with and without known COVID-19 positive human owners, information regarding the presence/absence of known COVID-19 in the household, was extracted as well.

### 2.3 Calculation of unprovided statistics

Some studies did not provide all the statistics to extract. Therefore, where possible, missing statistics were calculated based on other data reported in the studies. These included the number of positive cases, calculated based on the prevalence rate and overall sample size, and the 95% CI of the prevalence, calculated based on the number of positives and overall sample size using Wilson's method for proportions in Python (version 3.9).

### 2.4 Data visualization

Prevalence estimates were visualized according to their study periods to reflect the COVID-19 pandemic timeline, stratified by species (dogs or cats), setting (with or without known COVID-19 positive human owners), detection method (molecular or serological), and country where the study was conducted. The packages ‘tidyverse’ and ‘stringr’ for dataset preparation, and ‘tidyverse’ and ‘ggrepel’ for plotting, were used for data visualization in R (version 4.0.3) [32].

### 2.5 Meta-analysis

When the number of reported estimates for the molecular or serological prevalence in dogs or cats with or without known COVID-19 positive human owners was larger than 10, a meta-analysis was performed to generate pooled prevalence estimates of these studies using generalized mixed-effects models with the package ‘meta’ in R (version 4.0.3), following the standard procedures and default settings proposed for this type of analysis [33].

## 3 Results

### 3.1 Data description

In total, 17 studies were selected and included in this review. Fourteen studies investigated cats and 13 investigated dogs, and 10 investigated both. Five studies reported molecular prevalence and 16 studies reported serological prevalence, and 4 of these reported both. All 17 studies started in 2020 and 2 of them ended in 2021 (the others started and ended in 2020), and were conducted in 8 European countries (Italy, France, Spain, Croatia, Germany, the Netherlands, UK and Poland), 3 Asian countries (Iran, Japan and China) and the USA. Italy contributed with the biggest number of studies (*n* = 4).

The prevalence of SARS-CoV-2 in dogs and cats was determined in individual samples. Molecular methods were used in five studies, namely real-time quantitative reverse transcription PCR (qRT-PCR) [10,21,[34], [35], [36]]. Sixteen studies reported the use of one or multiple serological methods, including plaque reduction neutralization tests (PRNTs) [21], enzyme-linked immunosorbent assay (ELISA) [3,5,14,20,34,[37], [38], [39], [40]], virus neutralization test (VNT) [20,21,35,36,41,42], surrogate virus neutralization test (sVNT) [34], microneutralization test (MNT) [3], microsphere immunoassay (MIA) [4], indirect immunofluorescence assay (iIFA) [40] and an immunoassay based on paramagnetic beads, xMAP (Luminex Corp., https://www.luminexcorp.com) [38].

### 3.2 Prevalence in dogs and cats with known COVID-19 positive human owners

**Fig. 2:**
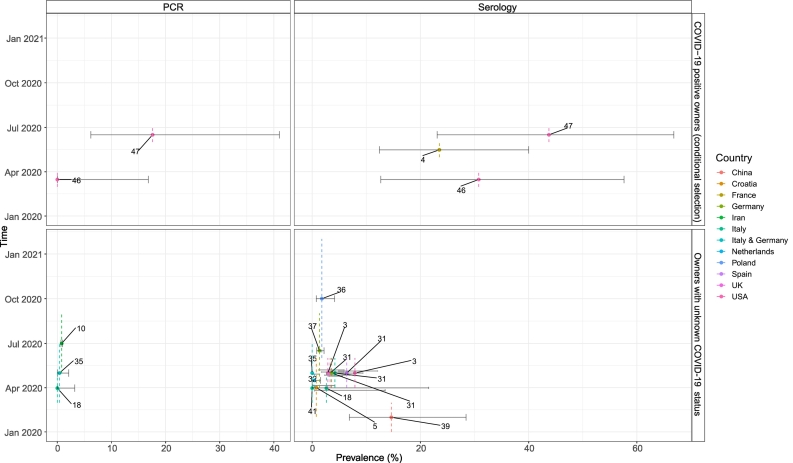
Molecular and serological prevalence estimates for SARS-CoV-2 in household cats with and without known COVID-19 positive owners from the different countries and sampling periods. Each point is positioned in the middle of the respective sampling period (y-axis) and assigned to a different color depending on the country of the study, showing the prevalence of SARS-CoV-2 infection (x-axis) among cats from each study as summarized in Table 2, Table 3. The whiskers around each point indicate the 95% CIs. The dotted line refers to the sampling period of the data in question. Text annotations per point are the reference of the studies. The upper category (’PCR’ and’Serology’) indicates the detection method, molecular or serological, respectively. The right axis indicates the study of cats with known (conditionally selected) COVID-19 positive owners (Table 2) and the study of cats without known COVID-19 positive owners (Table 3).

**Fig. 3:**
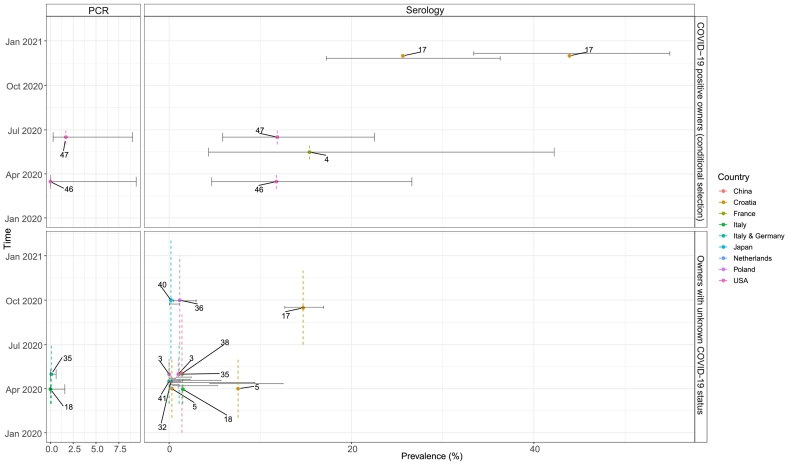
Molecular and serological prevalence estimates for SARS-CoV-2 in household dogs with and without known COVID-19 positive owners from the different countries and sampling periods. Each point is positioned in the middle of the respective sampling period (y-axis) and assigned to a different color depending on the country of the study, showing the prevalence of SARS-CoV-2 infection (x-axis) among cats from each study as summarized in Table 2, Table 3. The whiskers around each point indicate the 95% CIs. The dotted line refers to the sampling period of the data in question. Text annotations per point are the reference of the studies. The upper category (’PCR’ and’Serology’) indicates the detection method, molecular or serological, respectively. The right axis indicates the study of cats with known (conditionally selected) COVID-19 positive owners (Table 2) and the study of cats without known COVID-19 positive owners (Table 3).

Seven studies reported prevalence estimates in dogs and cats with exposure to known COVID-19 positive human owners. Two of these studies recruited pets before asking if owners had COVID-19, while the other five studies first selected COVID-19 positive households and then recruited their pets (conditional selection). Table 1 and Table 2 summarize the main characteristics of these two types of study. A meta-analysis could not be performed for molecular prevalence nor seroprevalence of SARS-CoV-2 infection in dogs or cats with known COVID-19 positive human owners due to the low number of point estimates available for pooling (Table 1, Table 2). The prevalence estimates for dogs and cats are shown in Fig. 2, Fig. 3, respectively.

**Table 1:** Overview of the studies reporting on the prevalence of SARS-CoV-2 infection in dogs and cats with known COVID-19 positive human owners.

Pet	Detection method	Sample type	Country	Study period	N total	N positives	Prevalence (%)	95% confidence interval	Reference
Cats	(N) xMAP	Serum	Italy	Mar’20-Jun’20	54	11	20.37	11.77 - 32.90⁎	[38]
	VNT	Serum	Italy	Mar’20-May’20	22	1	4.5	0.81 - 21.80⁎	[21]
Dogs	(N) xMAP	Serum	Italy	Mar’20-Jun’20	93	3	3.2	1.10 - 9.06⁎	[38]
	VNT	Serum	Italy	Mar’20-May’20	47	6	12.8	5.98 - 25.17⁎	[21]

⁎ Statistics not reported in the original studies and thus derived from available data using Wilson's method for proportions. VNT = Virus neutralization test. xMAP = Bead-based immunoassay. (N) = Nucleocapsid protein.

**Table 2:** Overview of the studies reporting on the prevalence of SARS-CoV-2 infection in dogs and cats conditionally sampled on the presence of known COVID-19 positive human owners.

Pet	Detection method	Sample type	Country	Study period	N total	N positives	Prevalence (%)	95% confidence interval	Reference
Cats	PCR	Respiratory; rectal	USA	Mar’20-Apr’20	19	0	0⁎	0 - 16.82⁎	[35]
	PCR	Respiratory; rectal	USA	Jun’20-Jul’20	17	3	17.6	6.19 - 41.03⁎	[36]
	(N,S1,S2) MIA	Serum	France	May’20-Jun’20	34	8	23.5	12.44 - 40.00⁎	[4]
	VNT	Serum	USA	Mar’20-Apr’20	13	4	30.8⁎	12.68 - 57.63⁎	[35]
	VNT	Serum	USA	Jun’20-Jul’20	16	7	43.8	23.1 - 66.82⁎	[36]
Dogs	PCR	Respiratory; rectal	USA	Mar’20-Apr’20	37	0	0⁎	0 - 9.41⁎	[35]
	PCR	Respiratory; rectal	USA	Jun’20-Jul’20	59	1	1.7	0.3 - 9⁎	[36]
	(N,S1,S2) MIA	Serum	France	May’20-Jun’20	13	2	15.4	4.33 - 42.23⁎	[4]
	(RBD) ELISA	Serum	Croatia	Dec’20	78	34⁎⁎	43.9	33.40 - 54.90⁎	[3]
	VNT	Serum	Croatia	Dec’20	78	20⁎	25.64	17.26 - 36.31⁎	[3]
	VNT	Serum	USA	Mar’20-Apr’20	34	4	11.8⁎	4.67 - 26.62⁎	[35]
	VNT	Serum	USA	Jun’20-Jul’20	59	7	11.9	5.87 - 22.52⁎	[36]

⁎ Statistics not reported in the original studies and thus derived from available data using Wilson's method for proportions.

⁎⁎ The number of positives was not reported in the original studies and did not result in an integer value when back-calculated using the reported prevalence rate x sample size. The number was therefore rounded down to the nearest integer. VNT = Virus neutralization test. PCR = Polymerase chain reaction. MIA = Microsphere immunoassay. ELISA = Enzyme-linked immunosorbent assay. (RBD) = Receptor-binding domain. (N) = Nucleocapsid protein. (S) = Spike protein.

Only seroprevalence estimates were reported in the two studies with no conditional sampling of dogs and cats on known COVID-19 positivity among the owners. There were contrasting findings when comparing dogs and cats from these two studies based on different detection methods in Italy during a similar sampling period [21,38]. Indeed, seropositivity was higher when cats were tested with xMAP and it was higher for dogs when they were tested with VNT.

The four studies with conditional sampling of dogs and cats on COVID-19 positivity among their owners were conducted in three countries (USA, France and Croatia) in slightly overlapping sampling periods. These studies reported a higher serological than molecular prevalence in both dogs and cats. The collection of different sample types (respiratory and rectal) was common in this study set-up for determining the molecular prevalence.

Two studies conducted in the USA used the same detection method and standard as advised by the US Department of Agriculture (USDA) to determine SARS-CoV-2 infection, molecularly or serologically, and the molecular and serological prevalence estimates of these two studies in dogs are very close with one another [35,36]. The seroprevalence estimates in France [4] were close to those in the USA, while the seroprevalence estimates in Croatia differed from those in the USA, although the detection methods were the same. Furthermore, the seroprevalence determined with VNT in pets conditionally sampled based on known COVID-19 positive human owners from Croatia was almost half of the one determined by ELISA in December 2020 [20].

### 3.3 Prevalence in dogs and cats without known COVID-19 positive human owners

Fourteen studies reported SARS-CoV-2 prevalence estimates in dogs and cats referred to veterinary clinics for various reasons, which may therefore include both healthy and sick animals. Eleven studies investigated cats, 10 investigated dogs, and 7 investigated both. Three studies reported molecular prevalence and 13 studies reported seroprevalence, and 2 studies reported both. Table 3 summarizes the main characteristics of those 14 studies, while Fig. 2, Fig. 3 show the prevalence estimates for cats and dogs, respectively. Contrary to pets with exposure to known COVID-19 positive owners, more studies with bigger sample sizes were available for pets with unknown exposure to COVID-19 positive owners. Generally, in this type of studies, prevalence determined with molecular methods was relatively low, <1%, and seroprevalence ranged from 0% to 14.7%. Twelve seroprevalence estimates in cats and 14 in dogs could be used in the meta-analysis to generate pooled seroprevalence estimates. These were 2.75% (95% CI: 1.56% to 4.79%) for cats and 0.82% (95% CI: 0.26% to 2.54%) for dogs. Further details of this analysis can be found in the Supplementary Information.

**Table 3:** Overview of the studies reporting on the prevalence of SARS-CoV-2 infection in dogs and cats with unknown COVID-19 positive human owners.

Pet	Detection method	Sample type	Country	Study period	N total	N positives	Prevalence (%)	95% confidence interval	Reference
Cats	PCR	Respiratory	Iran	May’20-Sep’20	124	1	0.81⁎	0.67 - 0.95	[10]
	PCR	Respiratory; rectal	Italy	Mar’20-May’20	116	0	0⁎	0 - 3.21⁎	[21]
	PCR	Respiratory	Italy; Germany	Mar’20-Jul’20	260	1	0.38	0.01 - 2.1	[34]
	VNT	Serum	UK	Apr’20-Jun’20	331	11	3.3	1.7 - 5.9	[42]
	VNT	Serum	Spain	Apr’20-Jun’20	360	23	6.4	4.1 - 9.4	[42]
	VNT	Serum	Germany	Apr’20-Jun’20	1136	48	4.2	3.1 - 5.6	[42]
	VNT	Serum	Italy	Apr’20-Jun’20	333	14	4.2	2.3 - 7.0	[42]
	PRNTs	serum	Italy	Mar’20-May’20	38	1	2.6	0.47 - 13.49⁎	[21]
	(N) ELISA	Serum	Poland	Jun’20-Feb’21	279	5	1.79	0.77 - 4.13	[14]
	(RBD) ELISA + VNT	Serum	Italy; Germany	Mar’20-Jul’20	24	0	0	0 - 1.38⁎	[34]
	(N) ELISA	Serum	USA	Apr’20-Jun’20	239	19	7.9	4.9 - 12.1	[5]
	(RBD) ELISA	Serum	USA	Apr’20-Jun’20	239	7	2.9	1.2 - 5.9	[5]
	MNT	Serum	Croatia	Feb’20-Jun’20	131	1	0.76	0.13 - 4.20⁎	[20]
	(RBD) ELISA	Serum	China	Jan’20-Mar’20	41	6	14.63	6.88 - 28.44⁎	[37]
	(N) xMAP	Serum	Italy	Mar’20-Jun’20	14	0	0⁎	0 - 21.53⁎	[38]
	(RBD) ELISA + iIFA	Serum	Germany	Apr’20-Sep’20	1173	16	1.36	0.84 - 2.20	[40]
	(RBD,S1) ELISA + VNT	Serum	Netherlands	Apr’20-May’20	500	2	0.4	0.01 - 1.55	[41]
Dogs	PCR	Respiratory; rectal	Italy	Mar’20-May’20	239	0	0⁎	0 - 1.58⁎	[21]
	PCR	Respiratory	Italy; Germany	Mar’20-Jul’20	877	1	0.1	0.02 - 0.63	[34]
	PRNTs	serum	Italy	Mar’20-May’20	133	2	1.5	0.41 - 5.32⁎	[21]
	(N) ELISA	Serum	Poland	Jul’20-Jan’21	343	4	1.17	0.45 - 2.96	[14]
	(S) ELISA + VNT	Serum	Japan	Jun’20-Feb’21	494	1	0.2	0.04 - 1.14⁎	[39]
	(RBD) ELISA + VNT	Serum	Italy; Germany	Mar’20-Jul’20	94	1	1.1	0.2 - 5.7	[34]
	(RBD) ELISA	Serum	Croatia	Jul’20-Dec’20	1069	157	14.69	12.69 - 16.93⁎	[3]
	(N) ELISA	Serum	USA	Apr’20-Jun’20	510	5	1	0.3 - 2.3	[5]
	(RBD) ELISA	Serum	USA	Apr’20-Jun’20	510	0	0	0 - 0.7	[5]
	MNT	Serum	Croatia	Feb’20-Jun’20	654	2	0.31	0.08 - 1.1⁎	[20]
	(S,N) ELISA	Serum	Croatia	Feb’20-Jun’20	172	13	7.56	4.47 - 12.5⁎	[20]
	(RBD) ELISA	Serum	China	Jan’20-Sep’20	851	12	1.41	0.81 - 2.45⁎	[50]
	(N) xMAP	Serum	Italy	Mar’20-Jun’20	37	0	0⁎	0 - 9.41⁎	[38]
	(RBD,S1) ELISA + VNT	Serum	Netherlands	Apr’20-May’20	500	1	0.2	0.01 - 1.24	[41]

⁎ Statistics not reported in the original studies and thus derived from available data using Wilson's method for proportions. VNT = Virus neutralization test. PCR = Polymerase chain reaction. MNT = Microneutralization test. ELISA = Enzyme-linked immunosorbent assay. PRNTs = Plaque reduction neutralization test. iIFA = Indirect immunofluorescence assay. xMAP = Bead-based immunoassay. (RBD) = Receptor-binding domain. (N) = Nucleocapsid protein. (S) = Spike protein.

Four studies conducted in four different countries used two different detection methods on the same samples to define positivity for SARS-CoV-2 in serological surveys, with three of them using a combination of ELISA and VNT to define positivity. Seroprevalence estimates differed in the same study when two different serological methods were used. A study in Minnesota, USA, used an ELISA based on the recombinant SARS-CoV-2 nucleocapsid protein and found that the seroprevalence was 7.9% (95% CI: 4.9% to 12.1%) in cats from April to June 2020. However, when the ELISA was based on the receptor-binding domain (RBD-ELISA), seroprevalence dropped to 2.9% (95% CI: 1.2% to 5.9%) in cats [5]. A study conducted in Croatia between 26 February and 15 June 2020, reported a seroprevalence of 0.31% (95% CI: 0.08% to 1.1%) among 659 dogs using MNT as the detection method [3]. This study also randomly selected 172 out of the 659 dogs to be tested with an ELISA based on the S and N antigens, thereby finding a higher seroprevalence of 7.56% (95% CI: 4.47% to 12.5%).

## 4 Discussion

We reviewed the available studies on the prevalence of SARS-CoV-2 in household dogs and cats with and without known COVID-19 positive owners, as determined by either molecular or serological methods, published from the beginning of the COVID-19 pandemic until January 2022. The majority of the reviewed studies looked at seroprevalence among pets with unknown COVID-19 positive owners, which was generally below 5%. Indeed, the meta-analysis showed a pooled seroprevalence of 2.75% (95% CI: 1.56% to 4.79%) for cats and 0.82% (95% CI: 0.26% to 2.54%) for dogs with unknown COVID-19 positive owners. This suggests that SARS-CoV-2 seroprevalence in the general pet population may also be at similar levels. SARS-CoV-2 seroprevalence in sheltered and stray cats has been reported to be similar: only 0.8% (95% CI: 0.1% to 3.0%) of sheltered cats tested positive using VNT in a study across 28 animal shelters in the Netherlands during the second wave of the pandemic [43], and 3.51% of stray cats had SARS-CoV-2 antibodies detected by RBD-ELISA in a study in the city of Zaragoza (Spain) between January and October 2020 [44]. Therefore, there seems to be limited variation in seroprevalence between pet cats and stray cats.

Comparing pets having known vs. unknown COVID-19 positive owners showed that seroprevalence in these two groups tends to diverge, with a generally higher prevalence in pets cohabiting with COVID-19 positive people (> 20% in cats and > 10% in dogs). This suggests that living with COVID-19 positive humans may be a risk factor for pets to become infected with SARS-CoV-2. However, people with COVID-19 are not the only potential infection source for their pets via interspecies transmission. Indeed, close contacts with other SARS-CoV-2 infected animals, such as minks, can also contribute to the spillover of SARS-CoV-2 to pets. A study investigating 101 cats living in or around infected mink farms (i.e., 89 stray cats and 12 household cats) in the Netherlands between April and November 2020, showed that 12 stray cats were molecularly and/or serologically positive for SARS-CoV-2, while no cats from the SARS-CoV-2 positive farmer's household were positive [45].

Except for the close contact with other infected humans or animals, there are also other recognized risk factors for pets to become infected with SARS-CoV-2. For instance, living in confined spaces might be one. Indeed, a study found a significantly higher seroprevalence in sheltered dogs vs. military working dogs living in a relatively closed environment [46]. Another study reported a higher seroprevalence in indoor vs. outdoor animals [38]. Gender is another potential risk factor for SARS-CoV-2 infection in dogs. A study found that male dogs are more likely to be positive than female dogs using ELISA [20]. The same study also reported that the age and breed of the dogs could be predisposing factors.

Some interventions seem useful to control the transmission of SARS-CoV-2 between pets and humans. A study investigated 54 cats and 42 dogs that were exposed to SARS-CoV-2 positive people (92 out of 96) or were close contacts of exposed animals (4 out of 96) in 4 shelters in the USA between May and July 2020 [47]. No cats or dogs tested positive by PCR and only one dog had detectable SARS-CoV-2 neutralizing antibodies (1:32 titer). The authors interpreted it as the result of the staff wearing appropriate personal protective equipment (PPE) and having limited contact time with the animals. Another study about pets in COVID-19 positive households also emphasized that the fewer contacts with owners, the lower risk for pets [35]. The pets were divided into a frequent (1 h) daily contact (with owners) group and a less frequent contact group, and seropositives could only be detected in the former group. Additionally, some studies detected SARS-CoV-2 on the surface of the pets' fur from the COVID-19 positive households [36,48]. Therefore, animal care workers can be recommended to wear face masks and gloves when they handle pets, and owners should keep their distance and avoid close contact with their pets when known to be infected with SARS-CoV-2.

This study has several limitations. Contrary to previous reviews, we differentiated between prevalence derived from screenings in the general pet population and prevalence among pets with known COVID-19 positive people in the household, and therefore compared the prevalence among pets with unknown exposure to COVID-19 positive owners (i.e., from the screenings) with the prevalence among pets exposed to the virus from their owners (whether or not pet sampling was conditional to COVID-19 positivity among humans). However, among those studies reporting prevalence among known COVID-19 positive households (i.e., those conditionally selected), the number of pets recruited from the same households, as well as the definition of COVID-19 positive households, differed. For instance, in a study involving 47 pets from 30 COVID-19 positive households, the pets were recruited from households where at least two people had COVID-19 [35]. However, in another study involving 76 pets from 39 COVID-19 positive households, the selection criteria were less strict, as these pets were recruited from households where at least one person had COVID-19 [36]. On the other hand, thirteen studies reporting prevalence from screenings recruited pets from those attending routine visits at veterinary clinics. Therefore, among the studies reporting prevalence from these screenings, we could not differentiate between healthy and sick animals. However, sick animals do not seem to be more prone to SARS-CoV-2 positivity compared to healthy animals, as a study focusing on sick cats found no SARS-CoV-2 positive cats among 83 sampled cats tested with PCR at a veterinary hospital in the state of Ohio, USA, during the summertime of 2020 [49]. Another limitation of the present review is that not all 17 studies collected their samples in the same phase of the COVID-19 epidemic in humans. Most of the selected studies spanned several months, which may include various stages of the COVID-19 epidemic where the prevalence can be very different over time. For example, between January and September 2020, the seroprevalence in dogs in Wuhan was 1.41%. The rates were observed to reach a peak in March at 7.89% and ended at 0% in June when the outbreak in Wuhan was under control [50]. The seroprevalence would be higher if we excluded the month of June. The geographical coverage of the selected studies is another source of sample heterogeneity. For example, one study may have collected samples from multiple places across the whole country, while another study may have only looked at one animal hospital in a specific city. Besides, the definition of SARS-CoV-2 positive pets can be varied among studies. Indeed, not all studies followed the guidelines of the OIE or the instructions of the USDA to define positive animals [35,36,48]. The detection methods in pets also vary. Some authors developed their own SARS-CoV-2 detection methods or used different methods to confirm the positives [5,34], while others used commercial methods [14]. Moreover, comparisons of data obtained using different serological techniques, with different levels of specificity and sensitivity, should be interpreted with caution, as for instance ELISA is known to be less specific that VNT due to possible cross-reactions with endemic coronaviruses of dogs and cats.

In conclusion, both seroprevalence and molecular prevalence of SARS-CoV-2 in household dogs and cats in the general pet population are generally below 5%, whereas they tend to exceed 10% when pets cohabit with known COVID-19 positive people in the household. This suggests interspecies transmission, with people having COVID-19 being a risk factor for SARS-CoV-2 transmission to their dogs and cats, and possibly *vice versa*. To improve our understanding of the potential epizootic implications of the COVID-19 pandemic, it is also important to establish global standards for SARS-CoV-2 detection in pets and to encourage researchers to use them consistently in prevalence studies.

## Declaration of Competing Interest

The authors declare that they have no known competing interests that could influence the work reported in this paper.

## Data Availability

All data are included in the article

## Appendix A Supplementary data

Supplementary material Image 1
