# Supplementary material for: When COVID-19 sits on people's laps: A systematic review of SARS-CoV-2 infection prevalence in household dogs and cats
Source: One Health. 2023 Feb 3;16:100497. doi: 10.1016/j.onehlt.2023.100497 (PMC9896854; doi:10.1016/j.onehlt.2023.100497)

# Supplementary Material

**Additional Figure 1.** Summary of the meta-analysis of serological prevalence estimates for SARS-CoV-2 in household cats with unknown COVID-19 positive owners. The meta-analysis of serological prevalence of cats in Table 3 is summarized below. The origins of the 14 seroprevalence estimates (proportions) used for fooling were indicated by reference.


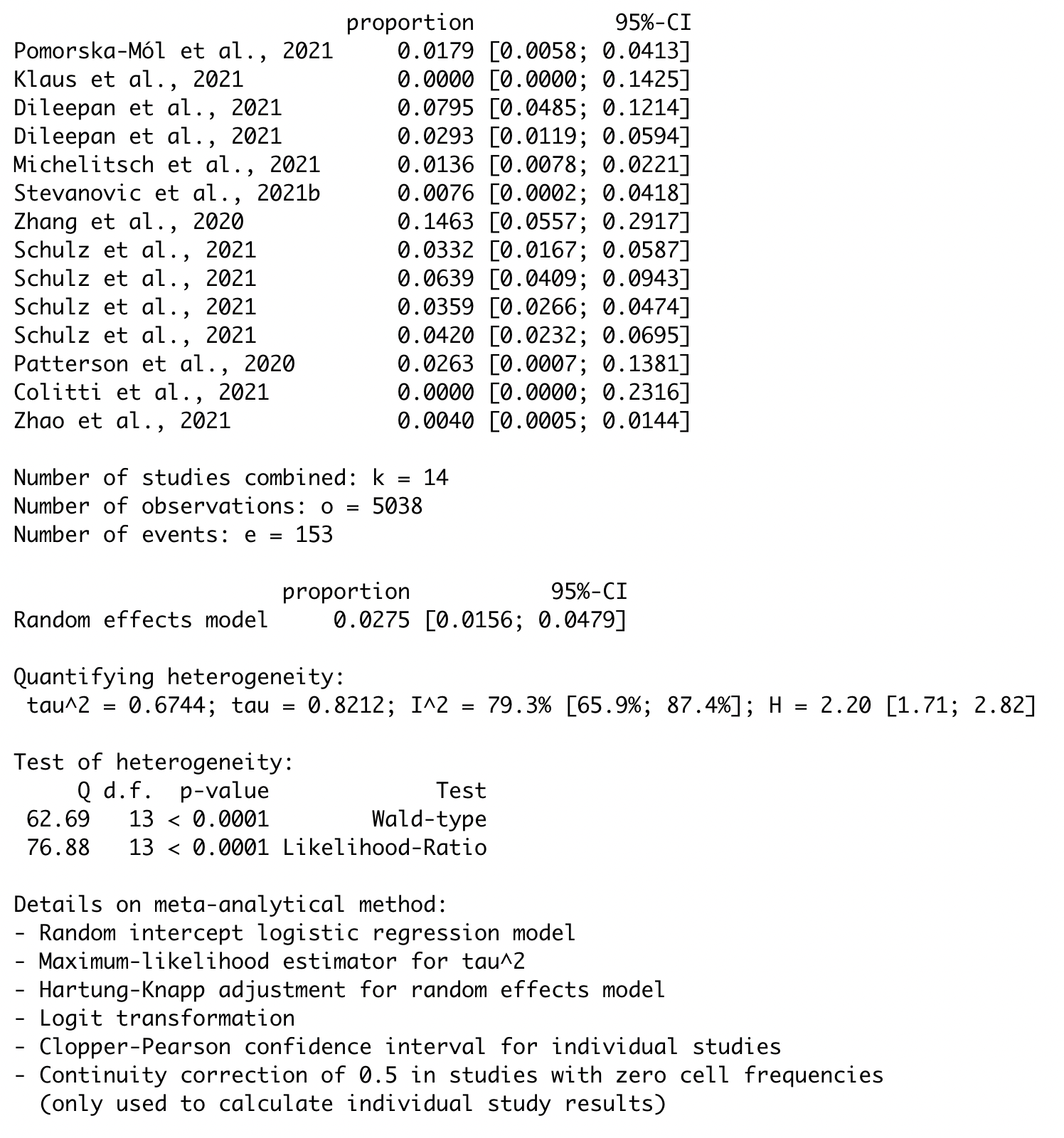


**Additional Figure 2**. Summary of the meta-analysis of serological prevalence estimates for SARS-CoV-2 in household dogs with unknown COVID-19 positive owners. The meta-analysis of serological prevalence of dogs in Table 3 is summarized below. The origins of the 12 seroprevalence estimates (proportions) used for pooling were indicated by reference.


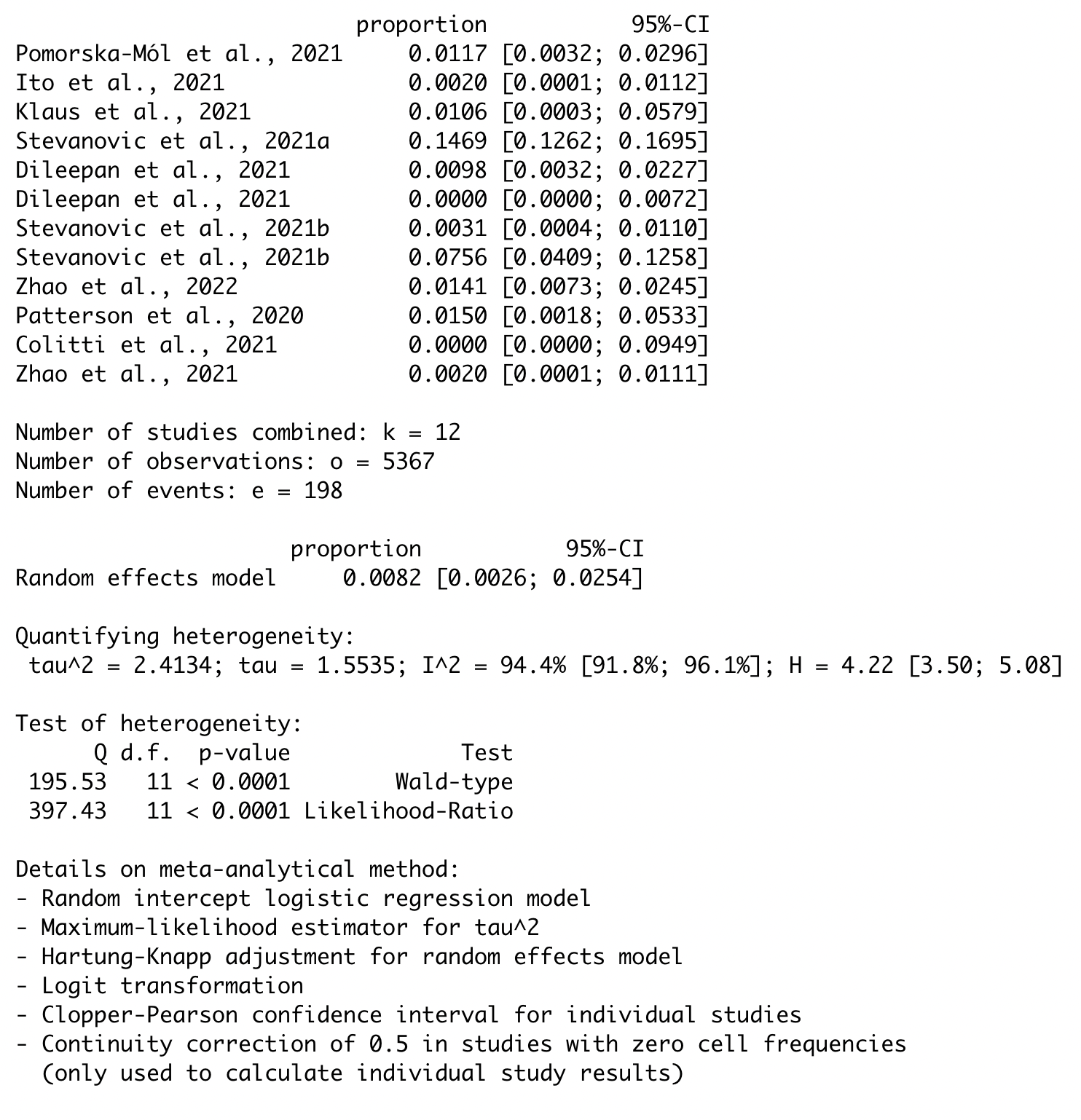

Supplement: Supplementary file 1 — Supplementary material [file mmc1.docx]
